# Enhanced Antibacterial Property of Sulfate-Doped Ag_3_PO_4_ Nanoparticles Supported on PAN Electrospun Nanofibers

**DOI:** 10.3390/molecules25061411

**Published:** 2020-03-19

**Authors:** Gopal Panthi, Md. Mehedi Hassan, Yun-Su Kuk, Ji Yeon Kim, Hea-Jong Chung, Seong-Tshool Hong, Mira Park

**Affiliations:** 1 Department of Biomedical Sciences and Institute for Medical Science, Jeonbuk National University, Jeonju 54907, Korea; gopalpanthi2003@gmail.com (G.P.); mehedibt07@gmail.com (M.M.H.); kiy9327@naver.com (J.Y.K.); 2 Korea Institute of Carbon Convergence Technology (KCTECH), Jeonju 54853, Korea; 3 Gwangju Center, Korea Basic Science Institute, Gwangju 61186, Korea; hjchung84@kbsi.re.kr

**Keywords:** electrospinning, SO_4_^2−^-Ag_3_PO_4_/PAN heterojunction, antibacterial, visible light, reactive oxygen species

## Abstract

Heterojunction nanofibers of PAN decorated with sulfate doped Ag_3_PO_4_ nanoparticles (SO_4_^2−^-Ag_3_PO_4_/PAN electrospun nanofibers) were successfully fabricated by combining simple and versatile electrospinning technique with ion exchange reaction. The novel material possessing good flexibility could exhibit superior antibacterial property over sulfate undoped species (Ag_3_PO_4_/PAN electrospun nanofibers). FESEM, XRD, FTIR, XPS and DRS were applied to characterize the morphology, phase structure, bonding configuration, elemental composition, and optical properties of the as fabricated samples. FESEM characterization confirmed the successful incorporation of SO_4_^2−^-Ag_3_PO_4_ nanoparticles on PAN electrospun nanofibers. The doping of SO_4_^2−^ ions into Ag_3_PO_4_ crystal lattice by replacing PO_4_^3−^ ions can provide sufficient electron-hole separation capability to the SO_4_^2−^-Ag_3_PO_4_/PAN heterojunction to generate reactive oxygen species (ROS) under visible light irradiation and enhances its antibacterial performance. Finally, we hope this work may offer a new paradigm to design and fabricate other types of flexible self-supporting negative-ions-doped heterojunction nanofibers using electrospinning technique for bactericidal applications.

## 1. Introduction

Bacteria constitute large group of single celled microorganisms that thrive in diverse environment. Along with the beneficial importance of bacteria, they also have many harmful effects on the human body, since some bacteria are the source of many serious diseases. Human activities including unimproved sanitation in developing areas, improper disposal of household waste, inadequate excreta disposal, and washing clothes near natural water sources are leading to an increasing risk of microbial contamination. Harmful and often drug-resistant bacteria, which are widespread in the environment can cause severe and deadly infections [1,2,3]. Hence, there is great deal of interest in developing antimicrobial agents with higher efficiency which would be promising for the economy. In this regard, different organic bactericides such as penicillin, streptomycin, tetracycline, etc., [4] are being used to kill bacteria; however, their use is limited due to serious issues of antimicrobial resistance and secondary pollution [5,6]. Besides, inorganic materials can also be the best alternative to organic bactericides because of their significant advantages, such as easy processing, thermal stability, being long lasting, and little drug resistance. Based on the action mechanism, inorganic materials are divided into semiconductor materials and metal-base materials. All semiconductor materials act as photocatalysts and are known to cause the formation of biologically reactive oxygen species (ROS), including hydroxyl/superoxide (OH^•^/O2^•−^) radicals in the presence of light. Thus, the produced ROS are responsible for the bactericidal activity of semiconductor materials but metal-based materials act as bactericidal agents without light assistance [7,8,9,10]. Among such semiconductor materials, silver phosphate (Ag_3_PO_4_) has drawn considerable attention as an antibacterial semiconductor material in recent years [11,12]. It is a narrow band gap semiconductor (2.36 eV), having the capability to generate ROS under visible light irradiation; however, it is noted that the application of Ag_3_PO_4_ as a photocatalyst is limited due to its poor chemical stability, when used without any sacrificial reagent in aqueous solution [13,14]. Furthermore, the agglomeration tendency of semiconductor photocatalyst when used in powder form is another serious issue, which reduces the effective surface area of particles, thereby decreasing their efficiency and making separation process more difficult after use. Therefore, after the breakthrough work carried out by Ye et al. [14], successive investigations have been performed to improve the photocatalytic efficiency of this semiconductor material with sufficient charge separation ability and stability by coupling it with other semiconductor/s [15,16], fabricating composites (with graphene [17,18], carbon nanotubes [19,20], non-metallic sorbent [21]) and doping suitable ions [22,23,24]. 

Semiconductor material doped with suitable ions could be an effective approach to design a photocatalyst with enhanced activity and stability [25]. Doping of suitable ions into the crystal lattice of semiconductor material provides significant capability to prevent the recombination of photogenerated electron-hole pairs and consequently improves the photocatalytic stability of the semiconductor materials. On that note, various attempts have been made to increase the photocatalytic performance of Ag_3_PO_4_ through cations doping into Ag_3_PO_4_ crystal lattice [26,27]. But the investigation on antibacterial activity of Ag_3_PO_4_ doped with suitable anions has been rarely reported. However, recently published report demonstrated that sulfur-doped Ag_3_PO_4_ can improve its photocatalytic activity on the basis of hybrid density-functional calculation [28] but the doping of sulfur into Ag_3_PO_4_ crystal lattice is not so easy by post-treatment process due to the strong P–O bond. Consequently, SO_4_^2−^ might be a suitable doping ion into Ag_3_PO_4_ crystal lattice by replacing PO_4_^3−^ since SO_4_^2−^ has smaller radius (0.218 nm) than that of PO_4_^3−^ (0.230 nm) [29,30]. The use of polymer electrospun nanofibers for incorporating photocatalyst nanoparticles through different fabrication methods is a promising strategy to provide enough reactive sites and avoid nanoparticles from being wasted during the separation process [31,32,33,34]. Because of extra-long one-dimensional structures, nanosized diameters and good flexibility, polymer electrospun nanofibers are considered to be good supports for immobilizing nanoparticles [35,36]. PAN is a widely used polymer for the fabrication of nanofibers using simple and versatile electrospinning techniques because of its good processability, environmental stability and low density. Therefore, PAN nanofibers are being used extensively as supports for photocatalyst nanoparticles [32,37]. 

Hence, to understand the combination of respective properties of SO_4_^2−^-doped Ag_3_PO_4_ nanoparticles and PAN electrospun nanofibers, we report the fabrication of the heterojunction of SO_4_^2−^-Ag_3_PO_4_/PAN electrospun nanofibers with enhanced antibacterial performance. To the best of our knowledge, such work has not been reported so far. For the convenience of description, different samples were hereinafter referred to as: pure PAN corresponding to pure PAN electrospun nanofibers, AP/PAN corresponding to sulfate undoped Ag_3_PO_4_/PAN electrospun nanofibers, and S-AP/PAN corresponding to SO_4_^2−^-doped Ag_3_PO_4_/PAN heterojunction nanofibers.

## 2. Results and Discussion

**Figure 1:**
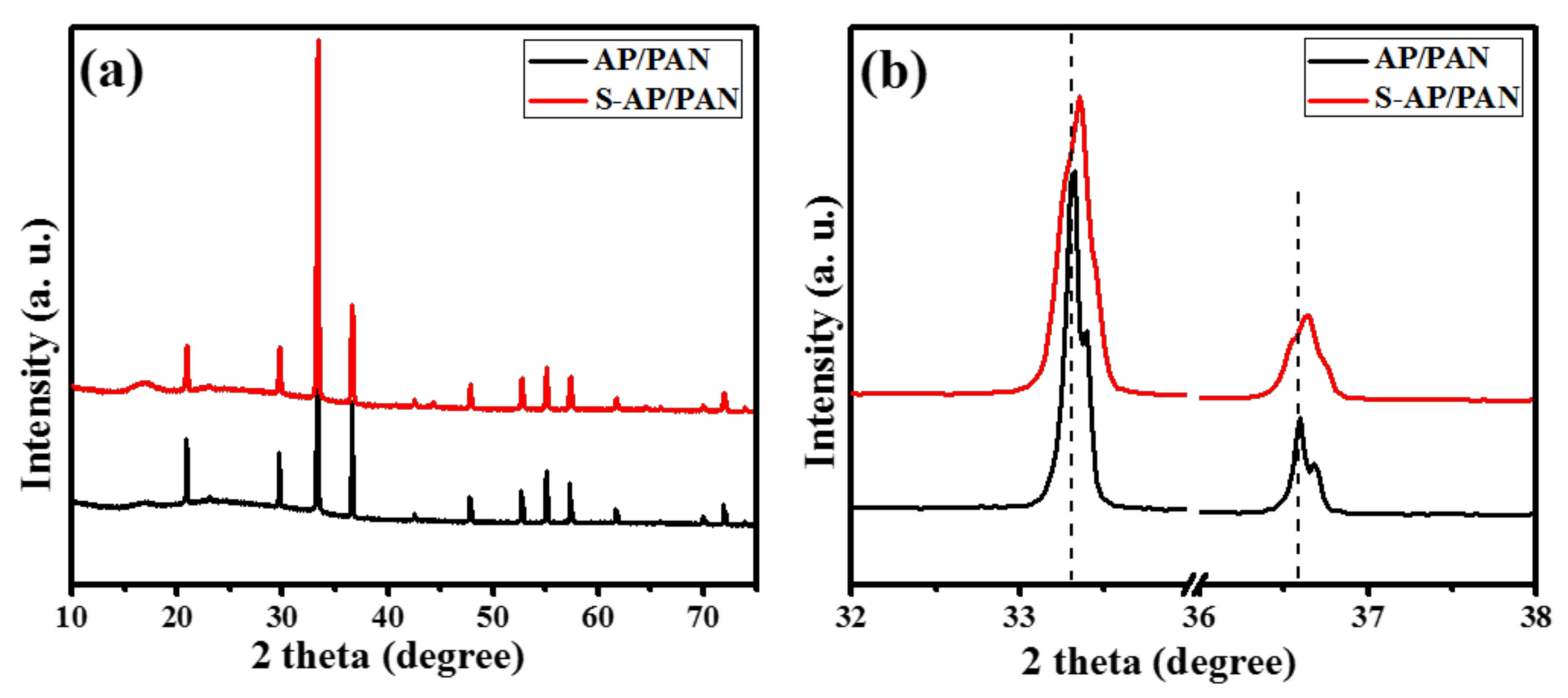
(**a**) XRD diffraction patterns; AP/PAN and S-AP/PAN and (**b**) magnified peaks of (210) and (211) crystal planes.

The crystalline structure and effect of SO_4_^2−^ doping on Ag_3_PO_4_ nanoparticles were investigated by XRD (Figure 1a). The diffraction peaks at 2*θ* of 20.8°, 29.7°, 33.3°, 36.5°, 42.4°, 47.7°, 52.6°, 55.0°, 57.2°, 61.6°, 65.7°, 70.0°, 71.8° and 73.7° in all formulations were attributed to the crystal planes of (110), (200), (210), (211), (220), (310), (222), (320), (321), (400), (411), (420), (421) and (332) of Ag_3_PO_4_, respectively (JCPDS card No: 06-0505). Besides, broad peaks centered at about 17° and 20°–23° in the composite nanofibers were assigned to the (100) and (110) crystallographic planes of PAN polymer, respectively [32]. The doping effect of SO_4_^2−^ into Ag_3_PO_4_ crystal lattice was investigated from the magnified XRD patterns of both samples (Figure 1b). As shown in the figure, the peaks corresponding to (210) and (211) crystal planes of S-AP/PAN are gradually shifted towards higher 2*θ* angles, which might be attributed to the decrease in crystal lattice constant due to the smaller ionic radius of SO_4_^2−^ than that of PO_4_^3−^ [30,38]. Therefore, replacement of PO_4_^3−^ by SO_4_^2−^ could allow the formation of SO_4_^2−^-doped Ag_3_PO_4_ nanoparticles.

**Figure 2:**
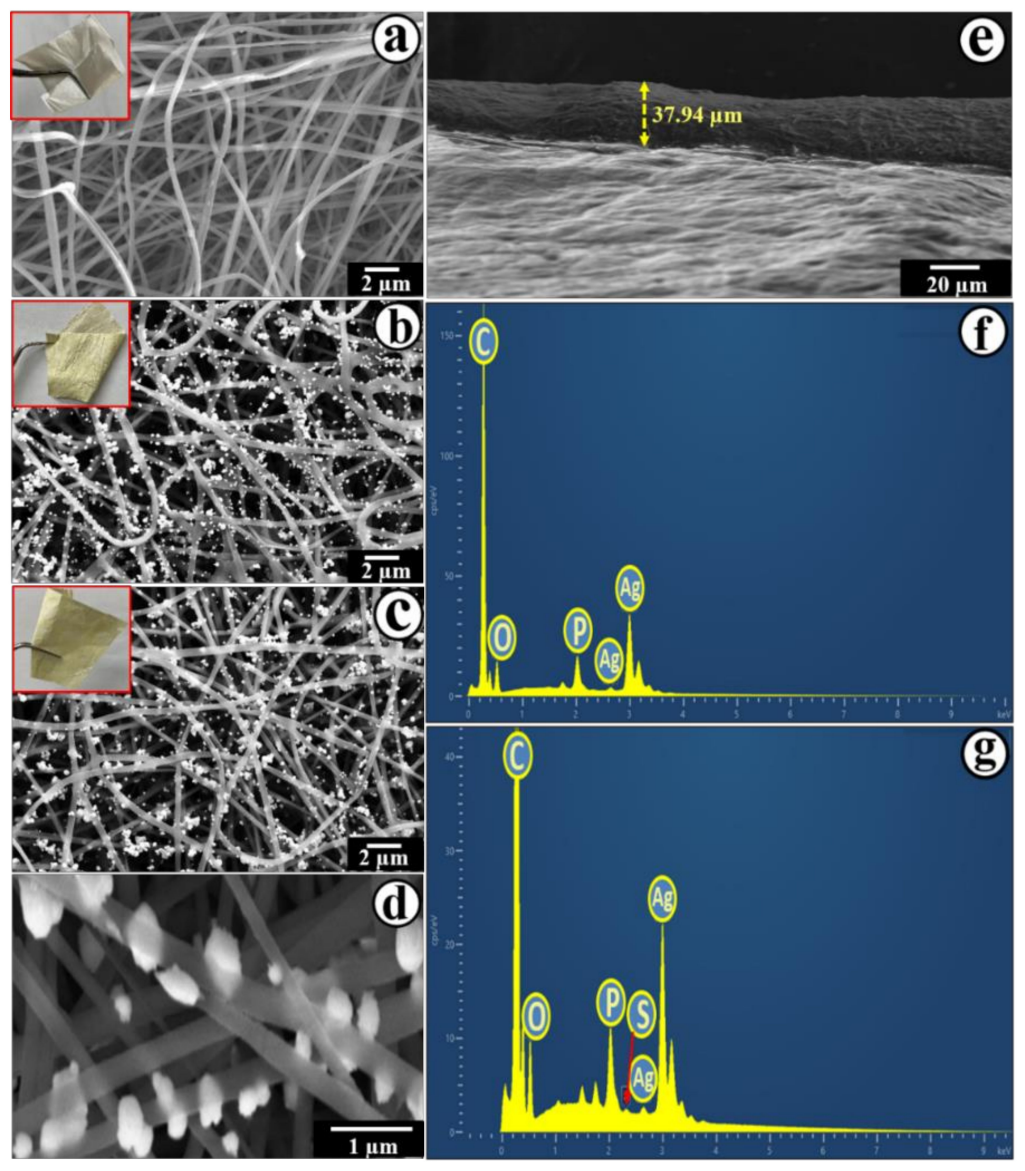
FESEM images; (**a**) Na_2_HPO_4_/PAN nanofibers, (**b**) AP/PAN, (**c**) S-AP/PAN, (**d**) high magnification image of S-AP/PAN and (**e**) cross section image of S-AP/PAN showing thickness of nanofibers mat. Panels (**f**) and (**g**) represent the corresponding EDS of (**b**) and (**c**), respectively. Insets; digital photograph of corresponding nanofibers.

**Figure 3:**
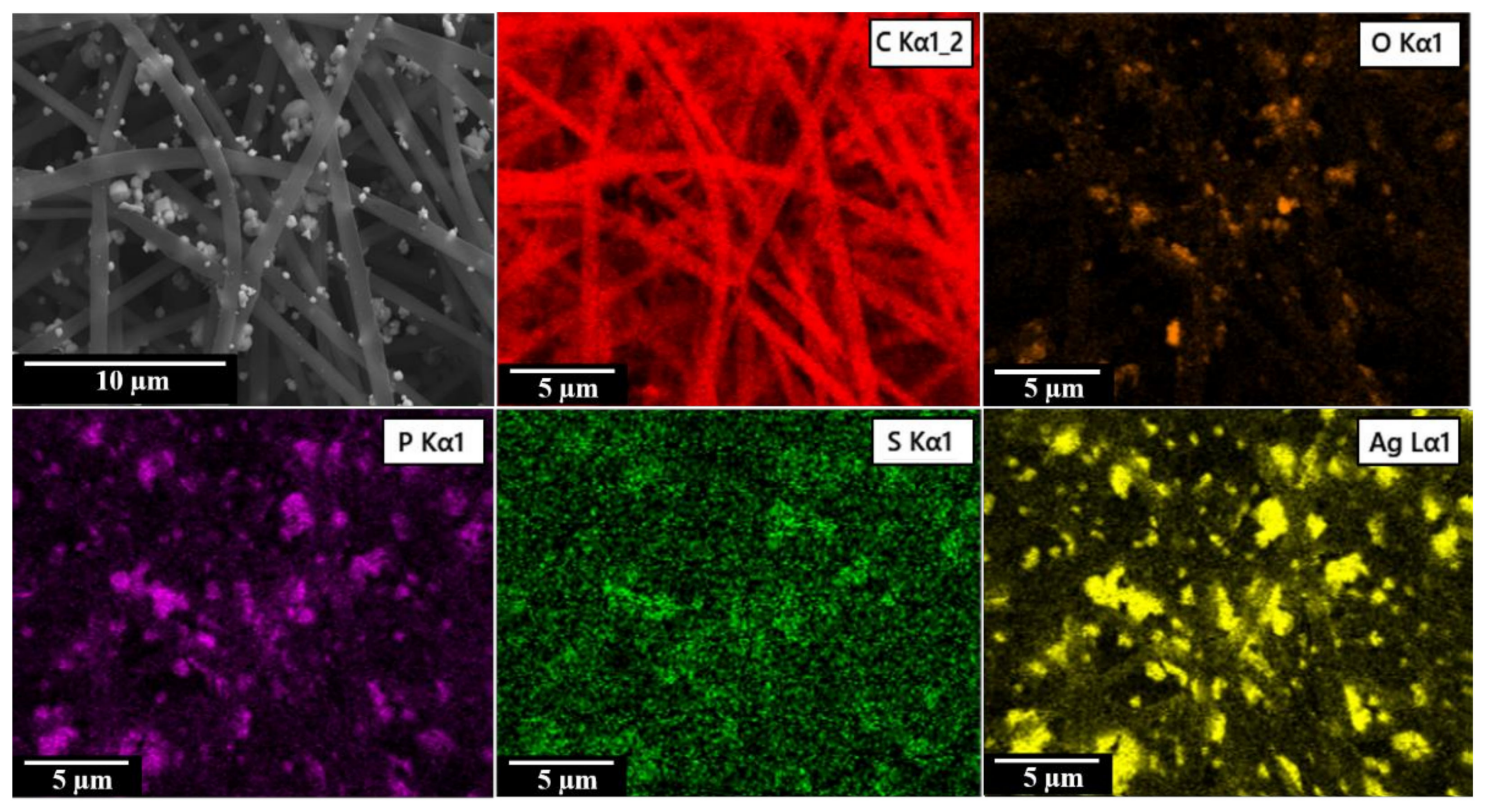
Elemental mapping of S-AP/PAN.

The morphological structure and distribution of nanoparticles on the surface of nanofibers were studied using FESEM (Figure 2). All formulations possess bead-free, continuous and randomly oriented nanofibers having an average diameter of 425 nm. Compared to Na_2_HPO_4_/PAN electrospun nanofibers (Figure 2a), the surface of AP/PAN nanofibers (Figure 2b) and S-AP/PAN nanofibers (Figure 2c) was no longer smooth due to the presence of secondary nanoparticles uniformly immobilized across the PAN nanofibers surface with some agglomerations. Figure 2d represents the high magnification image of S-AP/PAN showing the fiber/particles interface. As depicted in the figure, all the nanoparticles are well adhered to the nanofibers, so that these particles will not fall off the nanofibers in operational conditions. Furthermore, a cross section FESEM image was employed to measure the thickness of the nanofibers mat (Figure 2e). It appeared that the thickness of the S-AP/PAN nanofibers mat was estimated to be around 38 μm. The insets (Figure 2a–c) show the color change of Na_2_HPO_4_/PAN nanofibers from white to yellow indicating the growth of Ag_3_PO_4_ nanoparticles on the PAN nanofiber surface. Moreover, the elemental composition of composite nanofibers was investigated by FESEM-EDS. The spectra of AP/PAN displayed in Figure 2f indicated the presence of a considerable amount of C, O, P and Ag justifying the sample was composed of Ag_3_PO_4_ and PAN. Similarly, the presence of considerable amount of C, O, P, S, P and Ag as displayed in FESEM-EDS spectra of S-AP/PAN (Figure 2g) could confirm the doping of SO_4_^2−^ in Ag_3_PO_4_ nanoparticles without other impurity elements. The spatial distribution of elements was represented by the elemental mapping of S-AP/PAN (Figure 3). As shown in the figure, O, S, P and Ag are almost homogeneously distributed on the nanofibers, indicating the existence of SO_4_^2−^ in Ag_3_PO_4_ nanoparticles.

**Figure 4:**
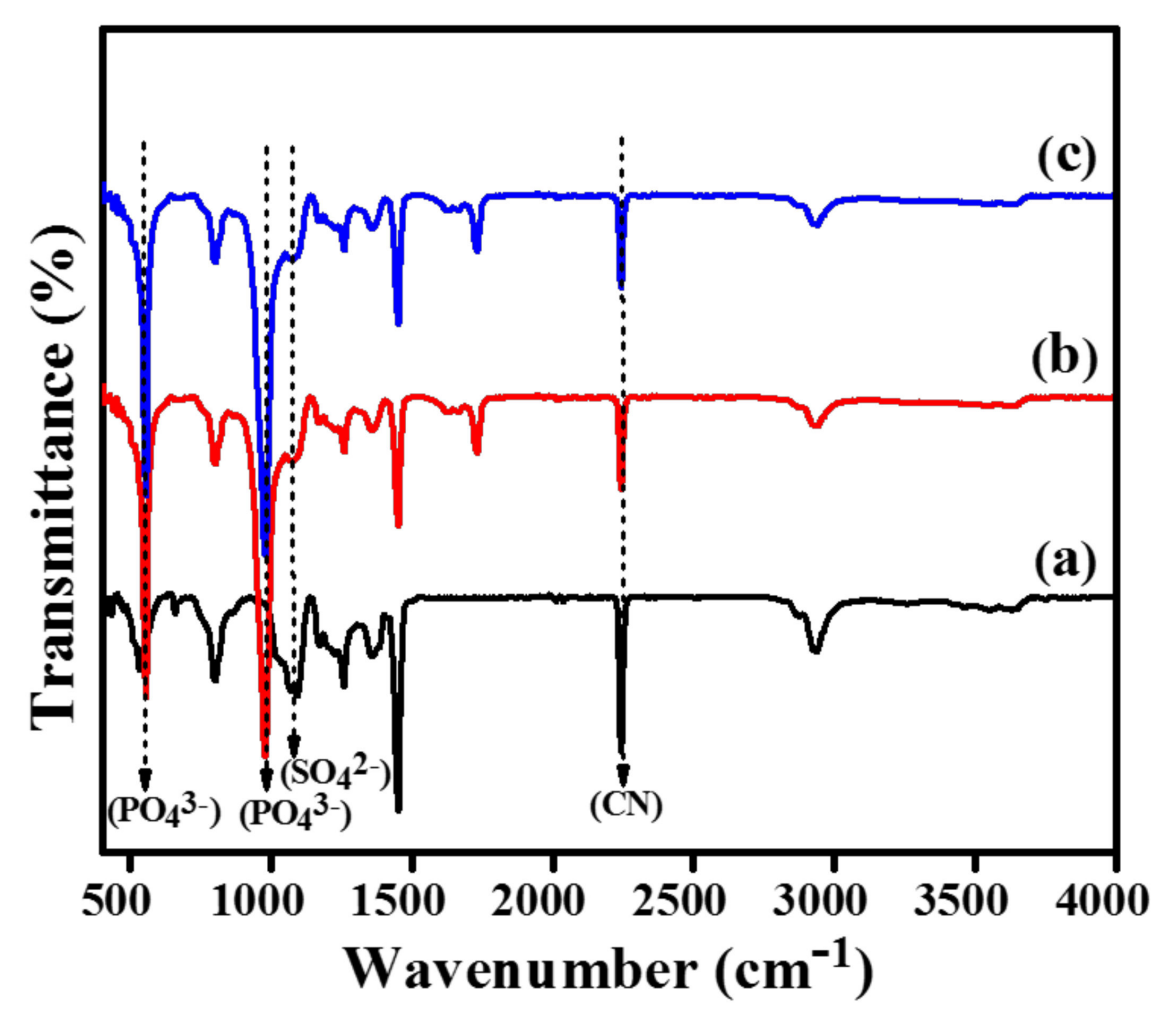
FTIR spectra; (**a**) pure PAN, (**b**) AP/PAN and (**c**) S-AP/PAN.

The FTIR spectra of three different samples are presented in Figure 4. The absorption band centered at about 2243 cm^-1^ in all samples is assigned to the nitrile group (C≡N) of PAN. Similarly, characteristics bands obtained in the regions 1220–1270 cm^−1^, 1350–1380 cm^−1^, 1450–1460 cm^−1^ and 2870–2931 cm^−1^ are assigned to the aliphatic CH group vibrations of different modes in the methylene group of PAN [39]. In all samples, the absorption bands attributed to the stretching vibration of H–O–H and bending vibration of O–H were located at about 1600 cm^−1^ and 3400–3500 cm^−1^, indicating the presence of physically absorbed water molecules [40]. In AP/PAN and S-AP/PAN, absorption bands located at about 550 cm^−1^ and 981 cm^−1^ could be assigned to the molecular vibration of PO_4_^3−^ [37,41]. But the absorption band corresponding to SO_4_^2−^ in S-AP/PAN, which locates at about 983 cm^−1^ [42,43], could not be visible due to overlapping with the absorption band of PO_4_^3−^. Hence, all the results obtained from FTIR analyses indicated the formation SO_4_^2−^-doped Ag_3_PO_4_ nanoparticles immobilized on PAN nanofibers.

**Figure 5:**
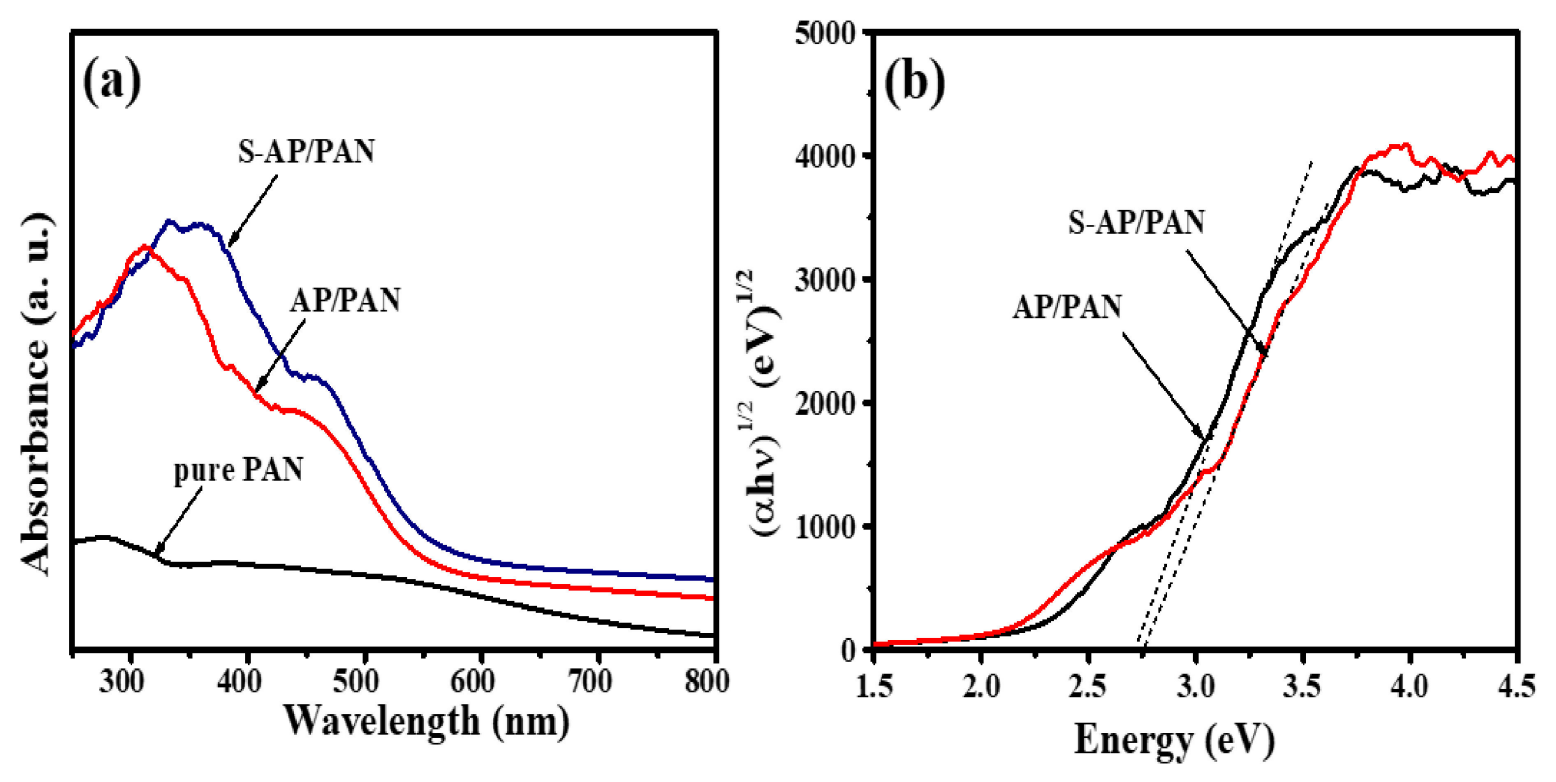
UV-vis diffusive reflectance spectra (**a**) and plot of (αhν)^1/2^ versus energy (hν) of as prepared photocatalysts (**b**).

In order to determine the light absorption behavior, UV-vis diffusive reflectance spectra (DRS) of different samples were measured and results are plotted in Figure 5. As shown in Figure 5a, two absorption bands ranging from 200–350 nm were observed in the DRS of pure PAN. After loading SO_4_^2−^-undoped/doped Ag_3_PO_4_ nanoparticles on the surface of PAN nanofibers, both the samples displayed obvious visible light absorption behavior, signifying their visible light absorption properties. The optical band gap energies (E_g_) of S-AP/PAN and AP/PAN were estimated using a plot of (αhν)^1/2^ versus energy (hν) [44]. As depicted in Figure 5b, the band gap energies of S-AP/PAN and AP/PAN were estimated to be 2.74 eV and 2.72 eV, respectively. This indicated that the band gap energy was increased slightly with SO_4_^2−^ doping.

**Figure 6:**
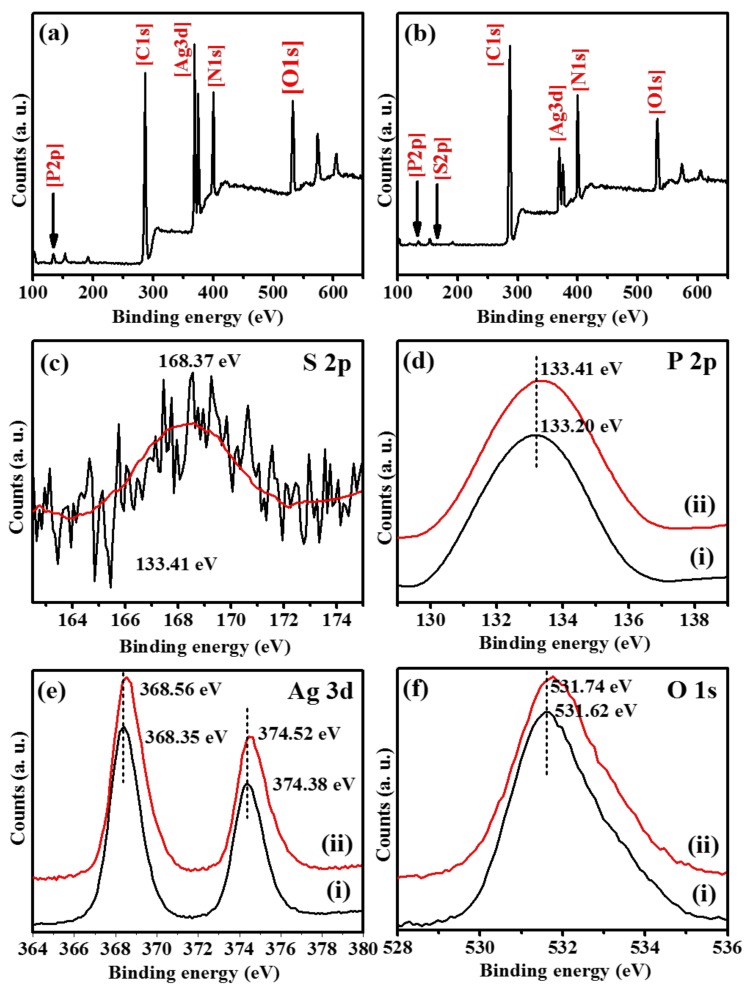
XPS spectra; survey spectra of AP/PAN (**a**) and S-AP/PAN (**b**). Panels (**c**–**f**) represent the high-resolution spectra of AP/PAN (i) and S-AP/PAN (ii).

The elemental composition and chemical state of elements in AP/PAN and S-AP/PAN were analyzed by XPS (Figure 6). The noticeable peaks of P 2p, C 1s, Ag 3d, N 1s and O 1s were clearly detected in the survey spectra of both samples (Figure 6a,b). The characteristics peaks corresponding to C 1s and N 1s which appeared around 284.6 eV and 397.6 eV, respectively, were from PAN (Figure 6a) [45]. Additionally, information regarding the specific nature of S, P, Ag and O was obtained from high resolution XPS spectra of both samples. In Figure 6c, the peak appeared at around 168.37 eV in high resolution spectrum of S 2p in S-AP/PAN is attributed to S^6+^ [46]. This result further confirmed that the incorporation of SO_4_^2−^ into the Ag_3_PO_4_ crystal lattice could occur during the synthesis process. The binding energies corresponding to P 2p and Ag 3d peaks of S-AP/PAN were found shifted to higher values compared to that of AP/PAN (Figure 6d,e). Such shifting might happen due to the doping of SO_4_^2−^, which results in decreased electron density around P and Ag because of higher electronegativity of S [47]. Similar behavior was observed for O 1s peak of S-AP/PAN compared to that of AP/PAN (Figure 6f). Therefore, all the results from XPS analysis suggested the incorporation of S in the form of SO_4_^2−^ in Ag_3_PO_4_ crystal lattice due to its strong interaction with the rest of the elements [25].

**Figure 7:**
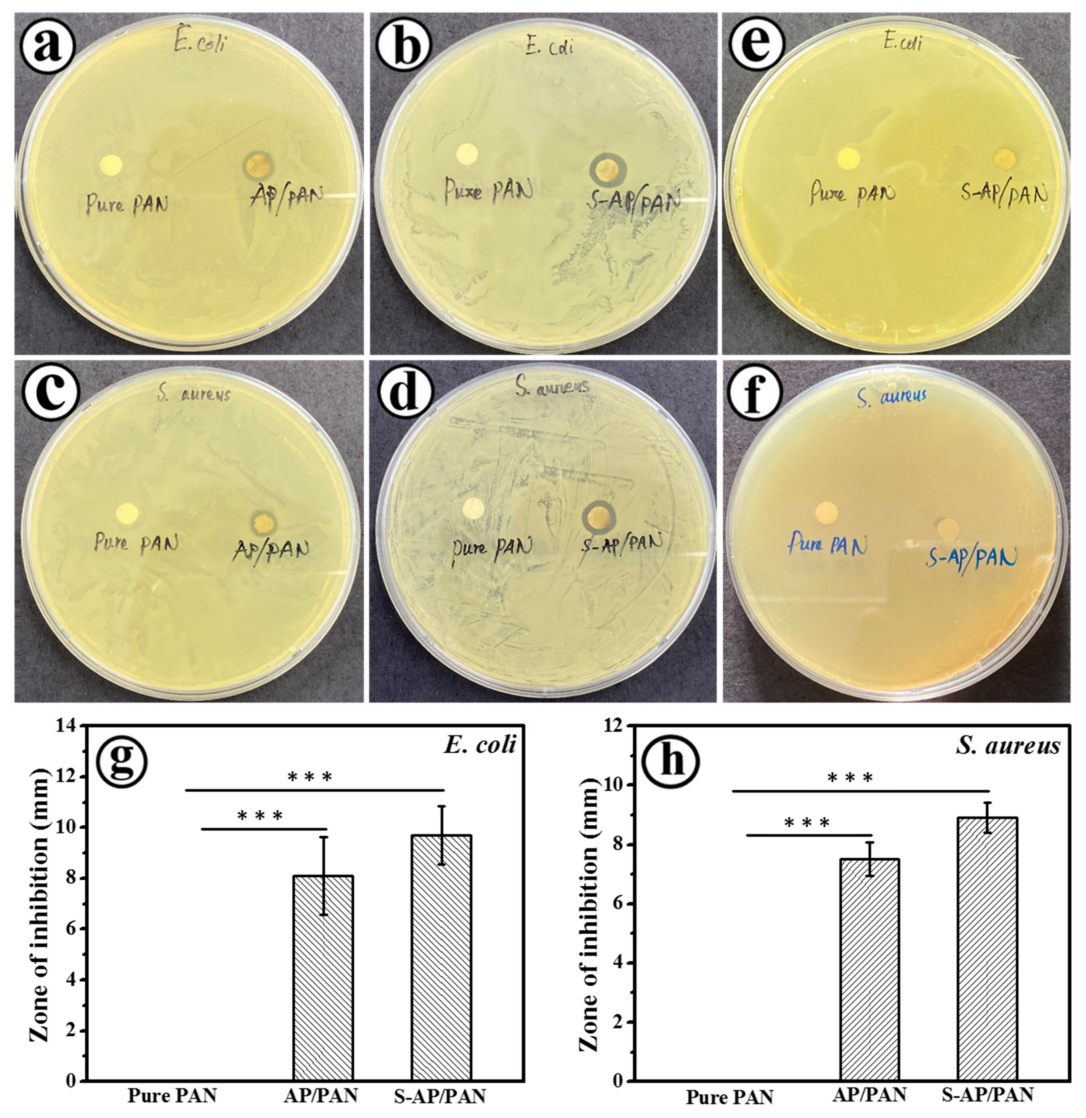
Disk diffusion test on *E. coli* (**a**,**b**) and *S. aureus* (**c**,**d**) by pure PAN, AP/PAN and S-AP/PAN. Panels (**e**) and (**f**) represent disk diffusion test on *E. coli* and *S. aureus*, respectively by pure PAN and S-AP/PAN in dark condition. AP/PAN and S-AP/PAN showed significant levels of zone of inhibition on *E. coli* (**g**) and *S. aureus* (**h**) compared to Pure PAN statistically assessed by one-way ANOVA using SPSS software. *p* < 0.05 considered as significant and represented as * *p* < 0.05, ** *p* < 0.01 and *** *p* < 0.001.

**Figure 8:**
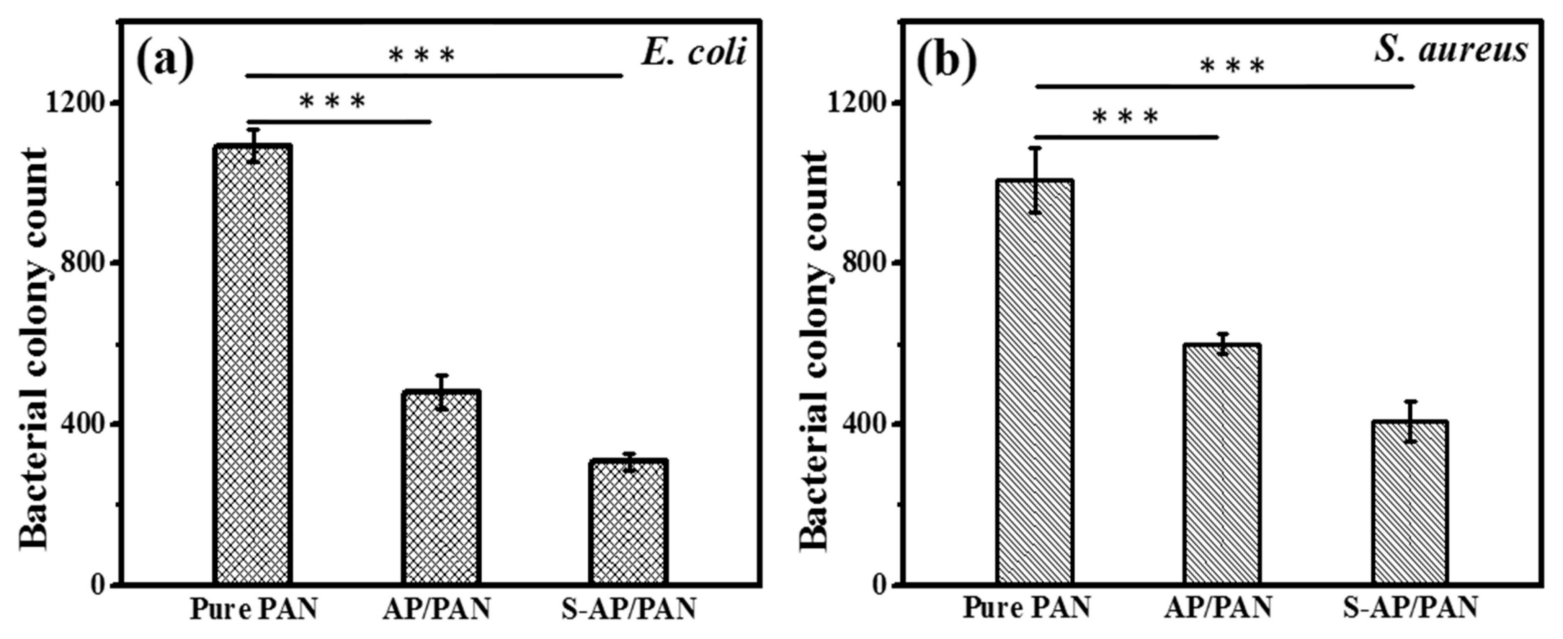
Number of colonies; (**a**) *E. coli* and (**b**) *S. aureus* grown on Tryptic soya agar plates after treatment with pure PAN, AP/PAN and S-AP/PAN. AP/PAN and S-AP/PAN showed significant levels of bacterial growth suppression activity compared to Pure PAN statistically assessed by one-way ANOVA using SPSS software. *p* < 0.05 considered as significant and represented as * *p* < 0.05, ** *p* < 0.01 and *** *p* < 0.001.

The antibacterial activities of pure PAN, AP/PAN and S-AP/PAN samples against *E. coli* and *S. aureus* were assessed by determining the diameter of inhibition zones, where pure PAN was used as a control. Antibacterial ability in the form of inhibition zones evaluated by the disk diffusion assay is shown in Figure 7. As shown in the figure, pure PAN showed no zone of inhibition indicating the lack of antibacterial activity for both Gram negative and Gram positive bacteria, while AP/PAN and S-AP/PAN clearly showed zones of inhibition. The zones of inhibition were 8.1 ± 1.52 mm and 9.7 ± 1.15 mm for *E. coli* for AP/PAN and S-AP/PAN, respectively (Figure 7a,b,g). Similarly, the zones of inhibition were 7.5 ± 0.57 mm and 8.9 ± 0.5 mm for *S. aureus* for AP/PAN and S-AP/PAN, respectively (Figure 7c,d,h). Figure 7e,f represent disk diffusion test on *E. coli* and *S. aureus*, respectively, using pure PAN and S-AP/PAN in dark condition. As depicted, no distinct zone of inhibition was observed, which signifies the absence of photogenerated ROS in dark condition. Furthermore, the antibacterial ability of pure PAN, AP/PAN and S-AP/PAN samples was tested by observing the survival of *E. coli* and *S. aureus* cells under day light (Figure 8). In this test, pure PAN was also used as control, which did not show any remarkable antibacterial activity but from the figure it is clear that S-AP/PAN had the lowest survivability of both types of bacteria compared to AP/PAN, however AP/PAN and S-AP/PAN showed significant levels of bacterial growth suppression activity compared to Pure PAN. Therefore, from these results it was seen that S-AP/PAN exhibited higher bacterial growth suppressing activity in both the methods and it can offer great promise to its application in the bactericidal activity. Moreover, the higher antibacterial efficacy of S-AP/PAN than that of S/PAN against *E. coli* compared to *S. aureus* might be related the cellular wall content differences between Gram-negative and Gram-positive bacteria [48,49].

The enhanced antibacterial performance of SO_4_^2−^-Ag_3_PO_4_/PAN heterojunction nanofibers can be explained with the role of SO_4_^2−^ as dopant. SO_4_^2−^-Ag_3_PO_4_, being a semiconductor material, produces electrons and holes when it is illuminated by visible light. The photogenerated electrons shift to the conduction band (CB) leaving behind holes in the valence band (VB). During this process, photoexcited electrons in CB react with dissolved oxygen in solution to produce O_2_^•−^ radicals, which further react with H^+^ to produce OH^•^ radicals. Similarly, photogenerated holes also react with H_2_O to produce OH^•^ radicals. Hence, thus produced OH^•^ radicals are responsible for photocatalytic antibacterial behavior of SO_4_^2−^-Ag_3_PO_4_/PAN heterojunction nanofibers [50]. It is believed that OH^•^ radicals assist the peroxidation of lipids in the outer cell wall of bacteria and damage cell organelles including DNA leading to cell death [50,51]. Most importantly, the doped SO_4_^2−^ ion plays an important role in trapping and transferring electrons. SO_4_^2−^-Ag_3_PO_4_ can receive additional electrons due to the presence of more valence electrons in S compared to P. Therefore, doping of SO_4_^2−^ into Ag_3_PO_4_ crystal lattice can enhance the concentration of photogenerated charges and inhibit the recombination of electron-hole pairs, thereby increasing the production of ROS, which is believed to be the main reason for the enhanced antibacterial activity of SO_4_^2−^-Ag_3_PO_4_ [28,52,53]. 

## 3. Materials and Methods

### 3.1. Chemicals

Polyacrylonitrile (PAN, MW-150000), Disodium hydrogen phosphate dihydrate (Na_2_HPO_4_.2H_2_O), Silver nitrate (AgNO_3_), Sodium sulfate (Na_2_SO_4_), were purchased from Sigma-Aldrich, USA. N, N-dimethylformamide (DMF) purchased from Daejung Chemicals, South Korea. All the chemicals were used as received without further purification.

### 3.2. Fabrication of Na_2_HPO_4_/PAN Nanofibers

First, 0.46 g of Na_2_HPO_4_ powder was ground with a mortar and pestle and dispersed in 14 ml of DMF using ultrasonication for 1 h. Then 1.5 g of PAN powder was added to the above dispersion with continuous magnetic stirring for 12 h in order to obtain homogeneous solution for electrospinning. Electrospinning of the prepared solution was carried out by loading into a plastic syringe provided with plastic micro-tip and using high voltage power supply at an applied voltage of 18 kV adjusting tip to collector distance 12 cm. The developing nanofibers were collected on a drum collector rotated at a constant speed by DC motor. All the experimental works were conducted at room temperature and atmospheric pressure. Afterward, the resulting electrospun nanofibers were vacuum dried at 70 °C for 12 h to remove the residual solvent.

### 3.3. Fabrication of Sulfate Doped Ag_3_PO_4_/PAN (SO_4_^2−^-Ag_3_PO_4_/PAN) Heterojunction Nanofibers

**Scheme 1:**
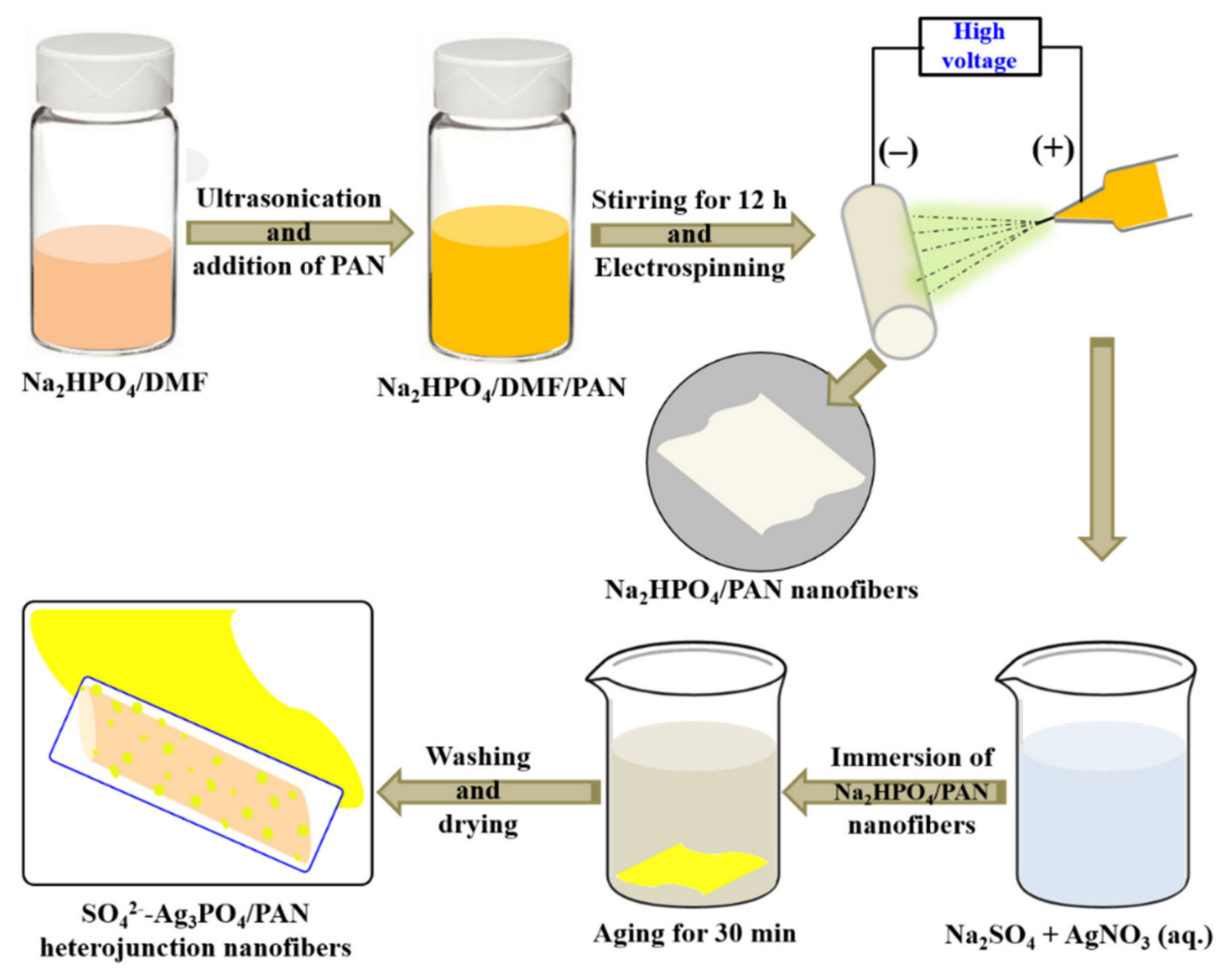
Schematic illustration for the fabrication of SO_4_^2−^-Ag_3_PO_4_/PAN heterojunction nanofibers.

0.1 g of Na_2_HPO_4_/PAN nanofibers mat was immersed in 500 ml of AgNO_3_ solution (0.02 M) containing 0.01 M of Na_2_SO_4_ for 30 min. The color of nanofibers mat was changed to yellow indicating the formation of SO_4_^2−^-Ag_3_PO_4_ nanoparticles immobilized on PAN nanofibers surface. For comparison, sulfate undoped Ag_3_PO_4_/PAN nanofiber mat was also fabricated without adding Na_2_SO_4_ under similar conditions. The resulting nanofibers mats were washed with copious amount of deionized water and dried at 60 °C for 6 h before characterization. The schematic for SO_4_^2−^-Ag_3_PO_4_/PAN heterojunction nanofibers fabrication is illustrated in Scheme 1.

### 3.4. Characterization

Information about phase and crystallinity of the samples were investigated using X-ray diffractometer (XRD, Empyrean, PANAlytical, Netherlands) with Cu Kα (λ = 1.540 Å) radiation over Bragg angles ranging from 10° to 80°. Morphological structure and distribution of nanoparticles on the surface of nanofibers were characterized using field emission scanning electron microscope (FESEM, GeminiSEM 500, Zeiss, UK) equipped with energy dispersive X-ray spectroscopy (EDS) to analyze the elemental composition of samples. Prior to characterization, samples were prepared by coating with platinum for 180 s using a Pt-coater (E-1030, Hitachi). Bonding configuration of the polymer with nanoparticles was characterized with the help of Fourier-transform infrared (FT-IR, FT/IR-4200, Jasco international Co., Ltd.) using attenuated total reflectance mode (ATR). Light absorption behavior of all samples was investigated from Uv-vis diffusive reflectance spectra (DSR) obtained from UV-vis spectrophotometer (Lambda 900, PerkinElmer, USA). Furthermore, the surface element composition analysis of SO_4_^2−^-doped/undoped samples was recorded using X-ray photoelectron spectroscopy (XPS, VG scientific Co., ESCA LAB MK II).

### 3.5. Antibacterial Activity Investigation

The antibacterial efficacy of the samples was assessed by using Gram negative *E. coli* (ATCC 25922) and Gram positive *S. aureus* (ATCC 4330) bacterial strains. Both disk diffusion and bacterial colony count tests were performed to determine growth suppressing action of pure PAN, AP/PAN and S-AP/PAN under day light conditions. In the disk diffusion test, Tryptic soya agar was used to perform the disk diffusion method to evaluate the antibacterial activity of the samples according to the previously described procedure [54]. Individual 0.5 McFarland sterile normal saline suspensions were prepared from freshly grown *E. coli* and *S. aureus* culture. A 100 µl cell suspension was plated in each tested petri dish and 5 mm disk of every samples were carefully dropped on top of the culture plates. All petri dishes were incubated at 37 °C for 12 h under day light and after incubation the zone of inhibitions were measured using ImageJ software. For comparison, the disk diffusion test was performed to evaluate the antibacterial activity of pure PAN and S-AP/PAN towards *E. coli* and *S. aureus* in dark conditions. The antibacterial effects of Pure PAN, AP/PAN and S-AP/PAN were further determined via bacterial colony count test on tryptic soya agar plates according to previous method with modification [55]. The fragments (3 cm × 5 cm) of test samples were embedded at the bottom of petri dish while making agar plates. A 0.5 McFarland cell suspension was serially diluted (10-fold dilution) 4 times in saline solution and, 100 µl aliquots of *E. coli* and *S. aureus* cell suspension were spread on each tested petri dish. Culture dishes were incubated at 37 °C for 12 h and the bacterial colonies were counted after incubation. All experiments were performed in triplicates, and statistical analysis was carried out using SPSS software. 

### 3.6. Statistical Analysis

All statistical data were expressed as the mean ± s. d., as indicated. Statistical significance was performed by one-way ANOVA using SPSS software [56]. *p* < 0.05 was considered as significant and represented as * *p* < 0.05, ** *p* < 0.01 and *** *p* < 0.001.

## 4. Conclusions

This was the first time that a heterojunction of sulfate-doped Ag_3_PO_4_ nanoparticles supported on PAN electrospun nanofibers was fabricated using the electrospinning technique followed by the ion exchange method. The antibacterial activity of the fabricated material was investigated on *E. coli* (Gram negative) and *S. aureus* (Gram positive) bacteria by disc diffusion and bacterial colony count methods under visible light. Experimental results showed that the flexible SO_4_^2−^-Ag_3_PO_4_/PAN heterojunction nanofibers (S-AP/PAN) can work as a bactericidal material with enhanced activity. 

## References

[1] Thompson J.M., Gündogdu A., Stratton H.M., Katouli M. (2013). Antibiotic resistant *Staphylococcus aureus* in hospital wastewaters and sewage treatment plants with special reference to methicillin-resistant Staphylococcus aureus (MRSA). J. Appl. Microbiol..

[2] Goldstein R.E.R., Micallef S.A., Gibbs S.G., Davis J.A., He X., George A., Kleinfleter L.M., Schreiber N.A., Mukherjee S., Sapkota S.W. (2012). Methicillin-resistant *Staphylococcus aureus* (MRSA) detected at four U.S. wastewater treatment plants. Environ. Health Perspect..

[3] Raita M.S., Iconaru S.L., Groza A., Cimpeanu C., Predoi G., Ghegoiu L., Badea M.L., Chifiriuc M.C., Marutescu L., Trusca R. (2020). Multifunctional hydroxyapatite coated with *Arthemisia absinthium* composites. Molecules.

[4] Fleming A. (1929). Classics in infectious diseases: On the antibacterial action of cultures of a penicillium with special reference to their isolation of *B. influenza*. Rev. Infect. Dis..

[5] Costanzo S.D., Murby J., Bates J. (2005). Ecosystem response to antibiotics entering the aquatic environment. Mar. Pollut. Bull..

[6] Watkinson A.J., Murby E.J., Kolpin D.W., Costanzo S.D. (2009). The occurrence of antibiotics in an urban watershed: From wastewater to drinking water. Sci. Total Environ..

[7] Ireland J.C., Klostermann P., Rice E.W., Clark R.M. (1993). Inactivation of *Escherichia coli* by titanium dioxide photocatalytic oxidation. Appl. Environ. Microbiol..

[8] Maness P.C., Smolinski S., Blake D.M., Huang Z., Wolfrum E.J., Jacoby W.A. (1999). Bactericidal activity of photocatalytic TiO_2_ reaction: Toward an understanding of its killing mechanism. Appl. Environ. Microbiol..

[9] Sun L., Qin Y., Cao Q.Q., Hu B.Q., Huang Z.W., Ye L., Tang X.F. (2011). Novel photocatalytic antibacterial activity of TiO_2_ microspheres exposing 100% reactive {111} facets. Chem. Commun..

[10] Panthi G., Barakat N.A.M., Al-Deyab S.S., El-Newehy M., Pandeya D.Y., Kim H.K. (2013). Interior synthesizing of ZnO nanoflakes inside nylon-6 electrospun nanofibers. J. Appl. Polym. Sci..

[11] Piccirillo C., Pinto R.A., Tobaldi D.M., Pullar R.C., Labrincha J.A., Pintado M.M.E., Castro P.M.L. (2015). Light induced antibacterial activity and photocatalytic properties of Ag/Ag_3_PO_4_ -based material of marine origin. J. Photochem. Photobiol. A.

[12] Thiyagarajan S., Singh S., Bahadur D. (2016). Reusable sunlight activated photocatalyst Ag_3_PO_4_ and its significant antibacterial activity. Mater. Chem. Phys..

[13] Liu W., Shen J., Yang X., Liu Q., Tang H. (2018). Dual Z-Scheme gC_3_N_4_/Ag_3_PO_4_/Ag_2_MoO_4_ ternary composite photocatalyst for solar oxygen evolution from water splitting. Appl. Surf. Sci..

[14] Yi Z., Ye J., Kikugawa N., Kako T., Ouyang S., Williams H.S., Yang H., Cao J., Luo W., Li Z. (2010). An orthophosphate semiconductor with photooxidation properties under visible-light irradiation. Nat. Mater..

[15] Xu J.W., Gao Z.D., Han K., Liu Y., Song Y.Y. (2014). Synthesis of magnetically separable Ag_3_PO_4_/TiO_2_/Fe_3_O_4_ heterostructure with enhanced photocatalytic performance under visible light for photoinactivation of bacteria. Acs Appl. Mater. Interfaces.

[16] Lin W., Zhang S., Wang D., Zhang C., Sun D. (2015). Ultrasound-assisted synthesis of high-efficiency Ag_3_PO_4_/CeO_2_ heterojunction photocatalyst. Ceram. Int..

[17] Yang X., Qin J., Jiang Y., Li R., Li Y., Tang H. (2014). Bifunctional TiO_2_/Ag_3_PO_4_/graphene composites with superior visible light photocatalytic performance and synergistic inactivation of bacteria. Rsc Adv..

[18] Liang Q., Shi Y., Ma W., Li Z., Yang X. (2012). Enhanced photocatalytic activity and structural stability by hybridizing Ag_3_PO_4_ nanospheres with graphene oxide sheets. Phys. Chem. Chem. Phys..

[19] Tian J., Li H., Xing Z., Wang L., Asiri A.M., Youbi A.O.A., Sun X. (2013). Facile synthesis of MWCNTs/Ag_3_PO_4_: Novel photocatalysts with enhanced photocatalytic activity under visible light. J. Nanoparticle Res..

[20] Wang Z., Yin L., Zhang M., Zhou G., Fei H., Shi H., Dai H. (2014). Synthesis and characterization of Ag_3_PO_4_/multiwalled carbon nanotube composite photocatalyst with enhanced photocatalytic activity and stability under visible light. J. Mater. Sci..

[21] Ma J., Zou J., Li L., Yao C., Kong Y., Cui B., Zhu R., Li D. (2014). Nanocomposite of attapulgite-Ag_3_PO_4_ for Orange II photodegradation. App. Catal. B Environ..

[22] Kim D.S., Cho Y.J., Park J., Yoon J., Jo Y., Jung M.H. (2007). (Mn, Zn) Co-doped CdS nanowires. J. Phys. Chem. C.

[23] Chandramohan S., Kanjilal A., Sarangi S.N., Majumder S., Sathyamoorthy R., Hong C.H., Som T. (2010). Effect of substrate temperature on implantation doping of Co in CdS nanocrystalline thin films. Nanoscale.

[24] Li J.X., Xu J.H., Dai W.L., Li H.X., Fan K.N. (2009). Direct hydro-alcohol thermal synthesis of special core-shell structured Fe-doped titania microspheres with extended visible light response and enhanced photoactivity. Appl. Catal. B.

[25] Zhang S.N., Zhang S.J., Song L.M. (2014). Super-high activity of Bi^3+^ doped Ag_3_PO_4_ and enhanced photocatalytic mechanism. Appl. Catal. B Environ..

[26] Xie Y.P., Wang G.S. (2014). Visible light responsive porous Lanthanum-doped Ag_3_PO_4_ photocatalyst with high photocatalytic water oxidation activity. J. Colloid Interface Sci..

[27] Yu H.C., Kang H.X., Jiao Z.B., Lü G.X., Bi Y.P. (2015). Tunable photocatalytic selectivity and stability of Ba-doped Ag_3_PO_4_ hollow nanosheets. Chin. J. Catal..

[28] Reunchan P., Umezawa N. (2015). Sulfur and silicon doping in Ag_3_PO_4_. J. Phys. Chem. C.

[29] Cao W., Gui Z., Chen L., Zhu X., Qi Z. (2017). Facile synthesis of sulfate-doped Ag_3_PO_4_ with enhanced visible light photocatalystic activity. Appl. Catal. B Environ..

[30] Roobottom H.K., Jenkins H.D.B., Passmore J., Glasser L. (1999). Thermochemical radii of complex ions. J. Chem. Educ..

[31] Tao R., Yang S., Shao C., Li X., Li X., Liu S., Zhang J., Liu Y. (2019). Reusable and flexible g-C_3_N_4_/Ag_3_PO_4_/Polyacrylonitrile heterojunction nanofibers for photocatalytic dye degradation and oxygen evolution. Acs Appl. Nano Mater..

[32] Panthi G., Park S.-J., Chae S.-H., Kim T.-W., Chung H.-J., Hong S.-T., Park M., Kim H.-Y. (2017). Immobilization of Ag_3_PO_4_ nanoparticles on electrospun PAN nanofibers via surface oximation: Bifunctional composite membrane with enhanced photocatalytic and antimicrobial activities. J. Ind. Eng. Chem..

[33] Gangemi C.M.A., Ludici M., Spitaleri L., Randazzo R., Gaeta M., D’Urso A., Gulino A., Purrello R., Fragalà M.E. (2019). Polyethersulfone mats functionalized with porphyrin for removal of para-nitroaniline from aqueous solution. Molecules.

[34] Panthi G., Park S.-J., Kim T.-W., Chung H.-J., Hong S.-T., Park M., Kim H.-Y. (2015). Electrospun composite nanofibers of polyacrylonitrile and Ag_2_CO_3_ nanoparticles for visible light photocatalysis and antibacterial applications. J. Mater. Sci..

[35] Panthi G., Barakat N.A.M., Khalil K.A., Yousef A., Jeon K.-S., Kim H.K. (2013). Encapsulation of CoS nanoparticles in PAN electrospun nanofibers: Effective and reusable catalyst for ammonia borane hydrolysis and dye photodegradation. Ceram. Int..

[36] Wang C., Wang J., Zeng L., Qiao Z., Liu X., Liu H., Zhang J., Ding J. (2019). Fabrication of electrospun polymer nanofibers with diverse morphologies. Molecules.

[37] Wang Q., Cui J., Li G., Zhang J., Li D., Huang F., Wei Q. (2014). Laccase immobilized on a PAN/Adsorbents composite nanofibrous membrane for catechol treatment by a biocatalysis/adsorption process. Molecules.

[38] Anisimov V.I., Zaanen J., Andersen O.K. (1991). Band theory and Mott insulators: Hubbard U instesd of Stoner. I. Phys. Rev. B.

[39] Zhang W., Liu J., Wu G. (2003). Evolution of structure and properties of PAN precursors during their conversion to carbon fibers. Carbon.

[40] Huang G.L., Zhu Y.F. (2007). Enhanced photocatalytic activity of ZnWO_4_ catalyst via fluorine doping. J. Phys. Chem. C.

[41] Miller L.M., Vairavamurthy V., Chance M.R., Mendelsohn R., Paschalis E.P., Betts F., Boskey A.L. (2001). In situ analysis of mineral content and crystallinity in bone using infrared micro-spectroscopy of the ν_4_ PO_4_^3−^ vibration. Biochim. Biophys. Acta.

[42] Sifontes Á.B., Cañizales E., Toro-Mendoza J., Ávila E., Hernández P., Delgado B.A., Gutiérrez G.B., Díaz Y., Cruz-Barrios E. (2015). Obtaining highly crystalline barium sulphate nanoparticles via chemical precipitation and quenching in absence of polymer stabilizers. J. Nanomater..

[43] Gomez M.A., Han B., Yao S., Chen Y., Li S., Zhang D., Wang S., Jia Y. (2019). A new and improved synthesis method for the formation of ZnFe-CO_3_ and ZnFe-SO_4_ hydrotalcites free from impurities. Appl. Clay Sci..

[44] Mane R.S., Lee W.J., Pathan H.M., Han S.H. (2005). Nanocrystalline TiO_2_/ZnO thin films: Fabrication and application to dye-sensitized solar cells. J. Phy. Chem. B.

[45] Yang T., Yang H., Zhen S.J., Huang C.Z. (2015). Hydrogen-bond-mediated in situ fabrication of AgNPs/Agar/PAN electrospun nanofibers as reproducible SERS subrtrates. Acs Appl. Mater. Interfaces.

[46] Cao W.R., Chen L.F., Qi Z.W. (2015). Microwave-assisted synthesis of Ag/Ag_2_SO_4_/ZnO nanostructures for efficient visible-light driven photocatalysis. J. Mol. Catal. A Chem..

[47] Wang D.H., Jia L., Wu X.L., Lu L.Q., Xu A.W. (2012). One-step hydrothermal synthesis of n-doped TiO_2_/C nanocomposites with high visible light photocatalytic activity. Nanoscale.

[48] Amato E., Diaz-Fernandez Y.A., Taglietti A., Pallavicini P., Pasotti L., Cucca L., Milanese C., Grisoli P., Dacarro C., FernandezHechavarria J.M. (2011). Synthesis, characterization and antibacterial activity against Gram positive and Gram negative bacteria of biomimetically coated silver nanoparticles. Langmuir.

[49] Taglietti A., Fernandez Y.A.D., Elvio Amato E., Cucca L., Dacarro G., Grisoli P., Necchi V., Pallavicini P., Pasotti L., Patrini M. (2012). Antibacterial activity of glutathione-coated silver nanoparticlesagainst Gram positive and Gram negative bacteria. Langmuir.

[50] Li Y., Zhang W., Neu J., Chen Y. (2012). Mechanism of photogenerated reactive oxygen species and correlation with the antibacterial properties of engineered metal-oxide nanoparticles. Acs Nano.

[51] Kiwi J., Nadtochenko V. (2005). Evidence for the mechanism of photocatalytic degradation of the bacterial wall membrane at the TiO_2_ interface by ATR-FTIR and laser kinetic spectroscopy. Langmuir.

[52] Jo W.J., Jang J.W., Kong K., Kang H.J., Kim J.Y., Jun H., Parmar K.P.S., Lee J.S. (2012). Phosphate doping into monoclinic BiVO4 for enhanced photoelectrochemical water oxidation activity. Angew. Chem. Int. Ed..

[53] Kung M.-L., Tai M.-H., Lin P.-Y., Wu D.-C., Wu W.-J., Yeh B.-W., Hung H.-S., Kuo C.-H., Chen Y.-W., Hsieh S.-L. (2017). Silver decorated copper oxide (Ag@ CuO) nanocomposite enhances ROS-mediated bacterial architecture collapse. Colloids Surf. B.

[54] Hombach M., Zbinden R., Böttger E.C. (2013). Standardisation of disk diffusion results for antibiotic susceptibility testing using the sirscan automated zone reader. BMC Microbiol..

[55] O’Toole D.K. (1983). Methods for the direct and indirect assessment of the bacterial content of milk. J. Appl. Bacteriol..

[56] IBM (2016). SPSS Statistics for Windows, Version 24.0..

